# Denervation-Induced Activation of the Standard Proteasome and Immunoproteasome

**DOI:** 10.1371/journal.pone.0166831

**Published:** 2016-11-22

**Authors:** Haiming M. Liu, Deborah A. Ferrington, Cory W. Baumann, LaDora V. Thompson

**Affiliations:** 1 Department of Physical Medicine and Rehabilitation, University of Minnesota, Minneapolis, Minnesota, United States of America; 2 Department of Ophthalmology and Visual Neurosciences, University of Minnesota, Minneapolis, Minnesota, United States of America; University of Louisville School of Medicine, UNITED STATES

## Abstract

The standard 26S proteasome is responsible for the majority of myofibrillar protein degradation leading to muscle atrophy. The immunoproteasome is an inducible form of the proteasome. While its function has been linked to conditions of atrophy, its contribution to muscle proteolysis remains unclear. Therefore, the purpose of this study was to determine if the immunoproteasome plays a role in skeletal muscle atrophy induced by denervation. Adult male C57BL/6 wild type (WT) and immunoproteasome knockout *lmp7*^*-/-*^*/mecl-1*^*-/-*^ (L7M1) mice underwent tibial nerve transection on the left hindlimb for either 7 or 14 days, while control mice did not undergo surgery. Proteasome activity (caspase-, chymotrypsin-, and trypsin- like), protein content of standard proteasome (β1, β5 and β2) and immunoproteasome (LMP2, LMP7 and MECL-1) catalytic subunits were determined in the gastrocnemius muscle. Denervation induced significant atrophy and was accompanied by increased activities and protein content of the catalytic subunits in both WT and L7M1 mice. Although denervation resulted in a similar degree of muscle atrophy between strains, the mice lacking two immunoproteasome subunits showed a differential response in the extent and duration of proteasome features, including activities and content of the β1, β5 and LMP2 catalytic subunits. The results indicate that immunoproteasome deficiency alters the proteasome’s composition and activities. However, the immunoproteasome does not appear to be essential for muscle atrophy induced by denervation.

## Introduction

In conditions of skeletal muscle atrophy (e.g., denervation), muscle mass is reduced, which ultimately impairs the muscle’s force producing capacity and ability to function [1–5]. Loss of muscle mass, also referred to as muscle atrophy, is primarily due to an imbalance between protein turnover with degradation exceeding synthesis. Two of the catabolic pathways responsible for shifting protein balance towards degradation include the autophagy-lysosome and ubiquitin-proteasome systems. Of these, the ubiquitin-proteasome system is responsible for degrading the myofibrillar proteins actin and myosin heavy chain [3,6,7], making it the main regulator of proteolysis in skeletal muscle.

One of the key components of ubiquitin-proteasome system is the standard proteasome. It is composed of a 20S core particle that consists of four stacked rings of seven subunits each. The outer rings of the 20S core contain constitutively expressed α-subunits, which associate with ATP-dependent regulatory PA700 complexes. The two inner rings contain the β-subunits. Within the β-rings, β1, β5 and β2 are the catalytic subunits and cleave after acidic, hydrophobic, and basic amino acid residues, respectively [8,9]. The standard proteasome is highly abundant in skeletal muscle and is responsible for degrading ubiquitinated proteins. Briefly, ubiquitin-conjugated proteins are deubiquitinated and transferred into the 20S proteasome core via PA700 [10] and degraded by the catalytic β-subunits. It is well established that several components and activities of the standard proteasome are increased in atrophying muscle [11–14]. However, the standard proteasome is not the only proteasome present in skeletal muscle. A variant of the standard proteasome, termed the immunoproteasome, also exists.

The immunoproteasome associates with PA28, a regulatory complex similar to PA700, but is ATP-independent. Another difference between the standard proteasome and immunoproteasome occurs within the β-rings. In the immunoproteasome, the β1, β5 and β2 subunits are replaced with the inducible subunits LMP2 (β1i), LMP7 (β5i) and MECL-1 (β2i), respectively. Under basal conditions, the immunoproteasome exists at low concentrations, roughly contributing 5% to the total proteasome population in skeletal muscle [15]. The immunoproteasome is well-known for its role in immune function, specifically generation of antigenic peptides [16] as part of immune surveillance [17–19]. Interestingly, under catabolic conditions such as aging [20,21], muscular dystrophy [22], and denervation [11], the immunoproteasome increases, which suggests a link between atrophy and the immunoproteasome may exist in skeletal muscle. A more extensive investigation is required to establish the validity of this assumption.

The purpose of this study was to determine if the immunoproteasome influences skeletal muscle proteolysis. To accomplish this, we determined how proteasome (i.e., standard proteasome and immunoproteasome) content and activity in skeletal muscle of both WT mice and *lmp7*^*-/-*^*/mecl-1*^*-/-*^ double knockouts (L7M1, immunoproteasome deficient) mice responded to denervation. Using this design, we were able to document how immunoproteasome deficient mice responded to denervation induced-atrophy and the effect it had on proteasome content and activity. We hypothesized that denervated muscle from L7M1 mice would be partially protected from atrophy.

## Materials and Methods

### Animals

Male 5–7 month-old C57BL/6 wild type (WT) and the double knockout *lmp7*^*-/-*^ *and mecl1*^*-/-*^ (L7M1) mice on a C57BL/6 genetic background [18] were used in the present study. All WT and L7M1 mice were randomly assigned to either an innervated (Day 0) or denervated (DN) group. Mice were fed *ad libitum* and maintained on a 14-hour light/ 10-hour dark cycle at 20°C. At the end of the study, mice were deeply anesthetized with an intraperitoneal injection of ketamine/xylazine (100 mg/kg ketamine, 10 mg/kg xylazine). While under anesthesia, the mice were weighed and dissected for tissue collection. The gastrocnemius (GAS) muscle was divided in half along the sagittal plane, flash frozen in liquid nitrogen and stored at -80°C until further use. Following the dissection, mice were euthanized by exsanguination. All procedures and protocols were approved by the Institutional Animal Care and Use Committee of the University of Minnesota.

### Tibial nerve transection

Mice from the DN group underwent tibial nerve transection on the left hindlimb for either 7 or 14 days, similar to that previously described [2,5,23]. Mice were anesthetized by inhalation of 2.5% isoflurane and prepped for surgery. Next, an incision (1 cm) was made from the sciatic notch to the knee followed by cutting through the hamstring muscle. The tibial nerve was then identified and separated from the peroneal and sural nerve branches at the area of the popliteal fossa. One knot was made (8–0 sterile silk suture) on the distal portion of the tibial nerve. To prevent re-innervation, the proximal portion of the tibial nerve (approximately 5 mm above the knot) was sutured to the biceps femoris muscle. A piece of the tibial nerve was removed between the suture site and the knot (≥3 mm segment). The muscle and skin-incision were closed by sutures and glued completely with vet-bond. After surgery, mice were given 0.1 ml Buprenorphine (0.03 mg/ml) for analgesia and monitored until they were ambulatory.

### Enriched proteasome preparation and protein extraction

An enriched proteasome preparation was modified from those previously described [21,22]. One half of the frozen GAS muscle was sealed in a pouch, dipped in liquid nitrogen and crushed using a hammer and pestle. The crushed muscle was homogenized in buffer A (0.1 M KCl, 20 mM MOPS, pH 7.0), centrifuged at 4,000 g for 20 minutes at 4°C and the supernatant was collected and saved. The remaining pellet was re-homogenized in buffer A and re-centrifuged at 4,000 g for 20 minutes at 4°C. The supernatant was then collected and combined with the supernatant that was initially saved. The combined supernatant fractions were then centrifuged at 1,180 g for 20 minutes at 4°C. The supernatant following this spin was collected and again centrifuged, but this time at 100,000 g for 16 hours at 4°C. Upon completion, the supernatant was discarded and the pellet containing proteasome was homogenized in buffer B (50 mM Tris-HCl, 5 mM MgCl_2_, 0.1% CHAPS, and 0.4% sucrose, pH7.5), stored at -80°C.

The other half of the GAS muscle was used for detecting protein content of autophagy marker microtubule associated protein light chain 3 (LC3). Briefly, a portion of GAS muscle (~35 mg) was homogenized in a RIPA buffer (Thermo Scientific, Rockford, IL) supplemented with a protease/phosphatase inhibitor cocktail (Thermo Scientific, Rockford, IL). The homogenate was centrifuged at 10,000 g for 15 minutes at 4°C and the supernatant was collected and saved at -80°C until further use.

### Western blotting

**Fig 1:**
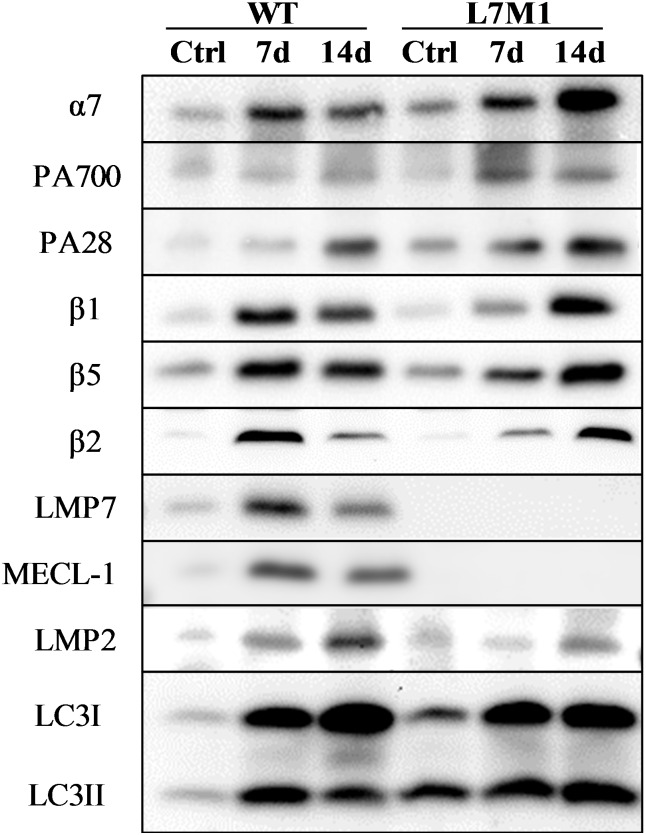
Representative Western blots. See Methods and Table 1 for experimental procedures and specific antibody information. These immunoblots show the gastrocnemius muscles of WT and L7M1 mice in control (innervated, Ctrl), and following 7 (7d) and 14 days (14d) of denervation. The selected antibodies are identified on the left.

Western blotting was performed to determine the protein content of the standard proteasome, immunoproteasome and LC3 (Table 1). Briefly, total protein content was first quantified with a bicinchoninic acid (BCA) assay (Thermo Scientific, Rockford, IL) using bovine serum albumin (BSA) as a standard. A portion of the muscle homogenate was diluted with reducing sample buffer (Thermo Scientific, Rockford, IL) and heated at 95°C for 4 minutes. Equal amounts of protein were then loaded onto a 13% sodium dodecyl sulfate (SDS)-polyacrylamide gel and separated by electrophoresis using a mini-vertical gel electrophoresis unit (BIO-RAD). The proteins were transferred to PVDF membranes using either a Trans-blot SD semidry transfer cell (BIO-RAD) at 14 V for 30 min (α7, β1, β5, β2 and LC3) or using a Mini Transfer-Blot Cell (BIO-RAD) at 110 V for 1 hour (all of the remaining proteins). After blocking in 5% HiPure Liquid Gelatin (Norland Products, Inc., Cranbury, NJ) or 5% non-fat dry milk in TBS/T at room temperature for 1 hour, membranes were incubated with primary antibodies overnight at 4°C. Following incubation, the membranes were washed and then probed with a goat anti-mouse (Thermo Fisher Scientific) or goat anti-rabbit HRP (BIO-RAD) secondary antibody. Prior to Western blotting, comparison of samples and conditions for each antibody were optimized to ensure that the reaction was within the linear ranges for signal intensity. See Table 1 for protein load and antibody information. The membranes were developed using SuperSignal West Dura Extended Duration chemiluminescence substrate (Pierce) and imaged using ChemiDoc XRS (BIO-RAD). Densitometry analysis was performed using Quantity One software (BIO-RAD). A GAS muscle from a WT mouse was used as a blot control to compare samples across different blots. Final protein content of each individual sample was expressed as a ratio to the blot control. Purified 20S proteasome (Boston Biochem, Cambridge, MA) and 20S immunoproteasome (Boston Biochem, Cambridge, MA) were used as positive controls for confirmation of the proteasome and immunoproteasome subunits. Fig 1 shows the representative Western blot for each protein from GAS muscles of WT and L7M1 mice at control, 7 and 14 days post-denervation.

**Table 1:** Antibodies used for Western blotting.

Antibody	Type	Host	Dilution	Protein load (μg)	Company for primary antibody	Secondary antibody dilution
**Proteasome subunit α7**	M	M	1:1000	4	Enzo Life Sciences, Farmingdale, NY	1: 16,000
**Proteasome 20S Y (β1)**	P	R	1:1000	10	Thermo Scientific, Rockford, IL	1: 16,000
**Proteasome 20S X (β5)**	P	R	1:1000	10	Thermo Scientific, Rockford, IL	1: 12,000
**Proteasome 20S Z (β2)**	P	R	1:500	30	Thermo Scientific, Rockford, IL	1: 20,000
**Proteasome subunit LMP2**	M	M	1:500	26	Enzo Life Sciences, Farmingdale, NY	1: 12,000
**Proteasome subunit LMP7**	P	R	1:1000	22	Enzo Life Sciences, Farmingdale, NY	1: 16,000
**Proteasome subunit MECL-1**	P	R	1:500	26	Enzo Life Sciences, Farmingdale, NY	1: 12,000
**19S (PA700)/Rpt1**	P	M	1:1000	15	Enzo Life Sciences, Farmingdale, NY	1: 10,000
**PA28α**	P	R	1:1000	15	Enzo Life Sciences, Farmingdale, NY	1: 10,000
**LC3B**	P	R	1:500	30	Novus Biologicals, Littleton, CO	1: 10,000

All antibodies were isotype IgG. Monoclonal (M), polyclonal (P), host species mouse (M), host species rabbit (R).

### Proteasome activity assay

Proteasome activities were determined using fluorogenic peptide substrates as previously described [22]. LLE-AMC (Proteasome Substrate II, Fluorogenic, EMDmillipore, Billerica, MA), LLVY-AMC (Proteasome Substrate III, Fluorogenic, EMDmillipore, Billerica, MA) and VGR-AMC (Bz-Val-Gly-Arg-AMC, ENZO) were used for caspase-, chymotrypsin-, and trypsin- like activities, respectively. Peptides were prepared as a 40 mM stock solution in DMSO and diluted in 50 mM Tris pH 7.8 buffer (final concentration: LLE– 200 uM; LLVY– 75 uM; VGR– 150 uM). Enriched proteasome preparations (4 ug/well for caspase- and chymotrypsin- activities; 7.5 ug/well for trypsin-like activity) were incubated in buffer (50 mM Tris pH7.8, 5 mM MgCl_2_, 20 mM KCl, and 1mM ATP) with or without 0.2 mM MG132 (Z-Leu-Leu-Leu-H, aldehyde, Peptides International, Louisville, KY), which is a proteasome inhibitor, for 30 minutes at 37°C before adding fluoropeptides. A negative control containing the fluoropeptide without any protein was used to determine the background signal. A serial dilution of 7-Amino-4-methylcoumarin (AMC, Sigma- Aldrich, St. Louis, MO) was used to generate a standard curve. Fluorescence was measured at 37°C in a CytoFluor 4000 Multiwell Plate Reader (Applied Biosystems, Foster City, CA) / Synergy^™^ HTX Multi-Mode Microplate Reader (BioTek, Winooski, VT) at a wavelength of 360/40 nm (excitation), 460/40 nm (emission) and a gain of 70 for 2 hours at 5-minute intervals. The activities were determined by comparing peptide fluorescence from samples with fluorescence of the standard curve of AMC.

### Histology

Cross-sectional area (CSA) of individual fibers from the GAS muscle was determined as previously described [24]. Briefly, a portion of the frozen GAS muscle was mounted with OCT (Tissue-Tek, Torrance, CA) and sliced at 10 μm using a Cryostat (Leica CM3050S, Nussloch, Germany) at -25°C. The muscle sections were dehydrated for 30 minutes, stained with hematoxylin and eosin, and imaged at 20x with a microscope (Nikon Eclipse E400). CSA was determined by circling 200–300 individual myofibers using ImageJ analysis software (National Institutes of Health, http://rsb.info.nih.gov/ij/).

### Statistical analysis

To determine the difference between strains across time a two-way ANOVA was utilized. If an interaction was detected (strain x DN days, *p* < .05), data was further analyzed by Fisher’s LSD *post hoc* test. For two of the proteins, LMP7 and MECL-1, a one-way analysis of variance (ANOVA) was performed to identify the time effect of denervation in the WT mice. An α-level of < 0.05 was used for all analyses. Values are presented in mean ± SEM. All statistical testing was performed using IBM SPSS version 22 software.

## Results

### GAS atrophy following denervation

**Fig 2:**
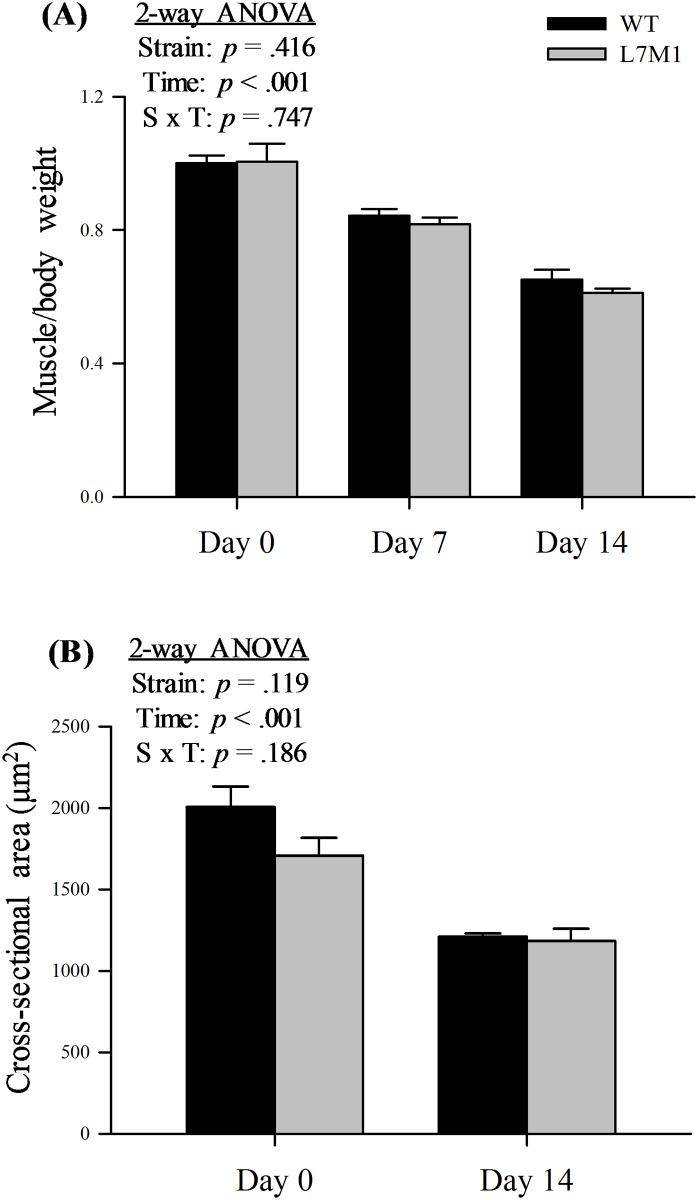
Atrophy of the GAS muscle and single fibers in WT and L7M1 mice. Two-way ANOVA was used to detect the effect of strain (WT vs. L7M1) and time (Day 0, 7, and 14). (A) Results of muscle/body weight are expressed relative to the WT Day 0. (B) CSA of individual fibers in GAS muscle (expressed as μm^2^). Statistical comparison showed a significant denervation (time) effect in both muscle/body weight and CSA (*p* < .001). No strain effect or interaction was detected in any of these measures. Values are mean ± SE. Sample size: for muscle/body weight: n = 7–10 per group; for CSA: n = 5–6 per group, 200–300 individual fibers analyzed per animal.

To assess the extent of atrophy in the GAS muscle after tibial nerve transection in WT and L7M1 mice, muscle weight (muscle/body weight) and cross-sectional area (CSA) were determined in each experimental group (Fig 2). Both WT and L7M1 mice exhibited a significant time-dependent decrease in normalized muscle weight after denervation. After 7 days of denervation, normalized muscle weight decreased 16% in WT and 19% in L7M1 compared to their baseline (day 0). At day 14, muscle loss continued, increasing to 35% and 39% in WT and L7M1, respectively. Importantly, no strain difference in the normalized muscle weight was detected (strain effect: *p* = .416). Consistent with these results, fiber CSA also significantly decreased following 14 days of denervation and did not differ between strains. Together, immunoproteasome deficiency does not appear to influence the extent of atrophy in GAS muscles after tibial nerve transection, at either 7 or 14 days post-surgery.

### Protein content of the proteasome

**Fig 3:**
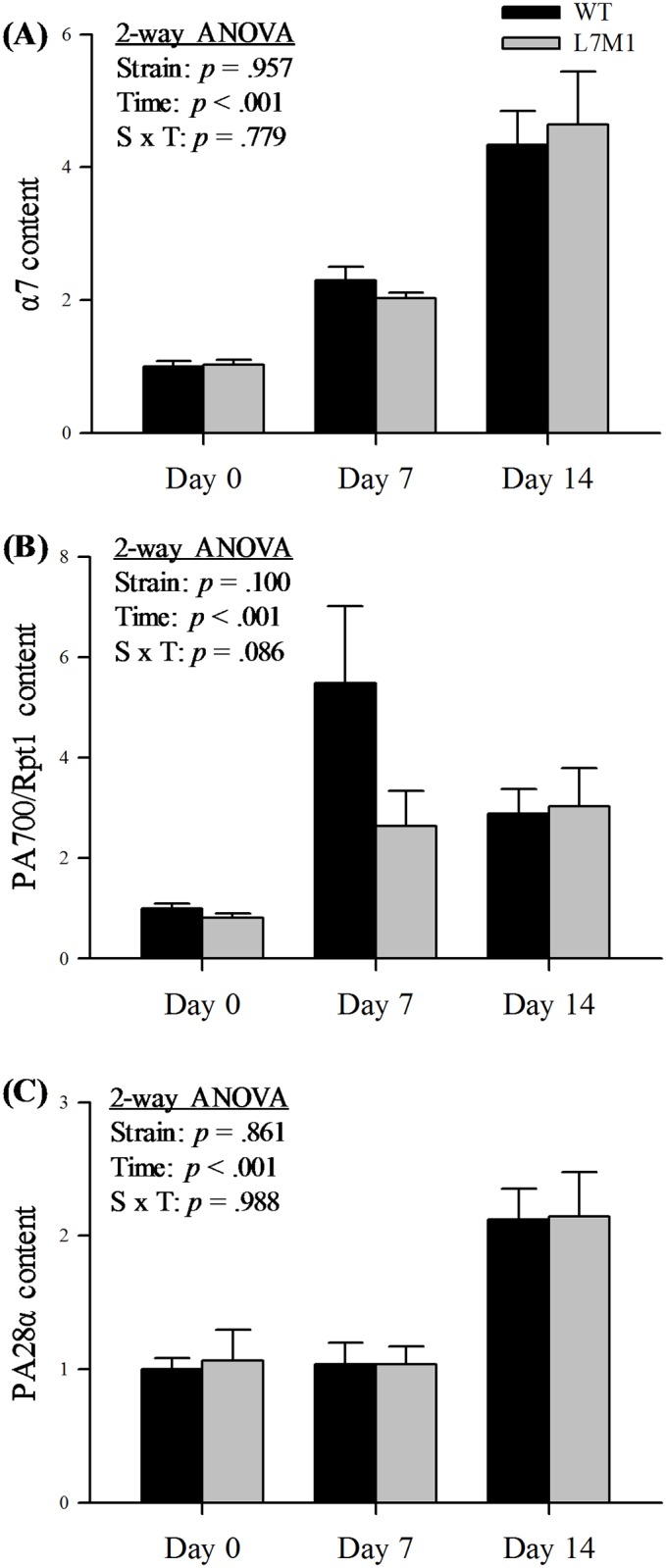
Protein content of α7 and proteasome activators in WT and L7M1 mice with denervation. (A-C) Results of densitometry are presented as the fold change compared to WT 0 Day for each respective protein. Each panel contains results of two-way ANOVA that were used to detect the effect of strain (WT vs. L7M1) and time (Day 0, 7, and 14) for (A) α7, (B) PA700/ Rpt1, and (C) PA28α (# indicates significant effect of strain/time/S x T interactions, *p* < .05). Statistical comparison showed a significant denervation (time) effect in all the proteins (*p* < .001). No strain effect was detected in any of these proteins. Values are mean ± SE. Sample size per group: n = 7–10. Representative Western blot for each protein is presented in Fig 1.

The α-subunits are constitutively expressed in the 20S proteasome and therefore is considered a reliable measure of the total proteasome [25]. With denervation, α7 content increased following denervation, but did not differ between strains (Fig 3A).

The multi-subunit regulatory complex PA700 binds to the 20S proteasome core to form a 26S proteasome. To estimate PA700 content, we measured Rpt1, one of the subunits of the complex. After denervation, PA700/Rpt1 content showed a time-dependent increase in both strains (Fig 3B). We also examined the content of PA28α, one of the subunits of the regulatory complex PA28, which binds to the immunoproteasome. We found that the content of PA28α increased significantly at day 14 when compared to day 0, and was similar between strains (Fig 3C).

**Fig 4:**
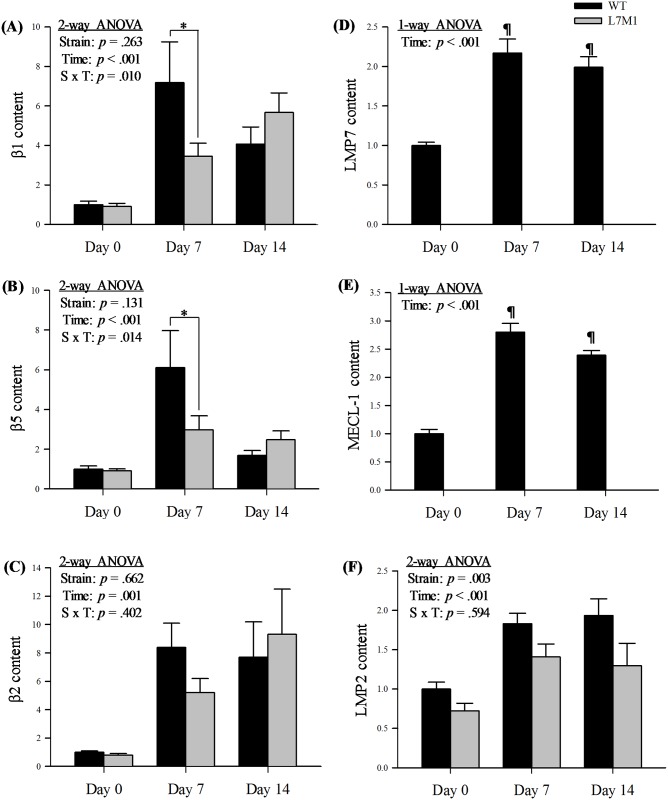
Protein content of standard catalytic and immunoproteasome subunits in WT and L7M1 mice with denervation. Results of densitometry are presented as the fold change compared to WT Day 0 for each respective protein. (A-C) Standard catalytic subunits. Each panel contains results of two-way ANOVA that were used to detect the effect of strain (WT vs. L7M1) and time (Day 0, 7, and 14) for (A) β1, (B) β5, and (C) β2 (# indicates significant effect of strain/time/S x T interactions, p < .05). When an effect of interaction was detected, a Fisher’s LSD post-hoc test was followed to compare differences between strains at each time point (* indicates L7M1 is significant different from WT at that given time point, p ≤ .05). Statistical comparison showed a significant denervation (time) effect in all the proteins above (p < .001). No strain effect was detected in any of these proteins. (A) β1 was significantly higher at day 7 in the WT compared to L7M1 (S x T, p = .01, LSD post-hoc test: p = .003). (B) β5 was significantly higher at day 7 in the WT compared to L7M1 (S x T, p = .01, LSD post-hoc test: p = .002). (D-F) Immunoproteasome subunits. For (D) LMP7 content and (E) MECL-1 content, the one-way ANOVA statistical comparison showed a significant denervation (time) effect in LMP7 and MECL-1 content (p < .001). # indicates significant time effect; ¶ indicates significant difference comparing to the 0d. (F) For LMP2 content: The two-way ANOVA result is shown in this panel (# indicates significant effect of strain/time, p < .05). Statistical comparison showed a significant denervation (time) effect (p < .001) and strain effect (p = .003) in LMP2 content. Values are mean ± SE. Sample size per group: n = 4–10. Representative Western blot for each protein is presented in Fig 1.

The β subunits responsible for the proteasome’s catalytic activities include the standard subunits (β1, β5, β2) and the inducible subunits (LMP2/β1i, LMP7/β5i, MECL-1/β2i). In general, all standard subunits increased in response to denervation in both strains (Fig 4). However, the denervated muscles from the WT mice exhibited a robust elevation in β1 and β5 content at day 7 compared to L7M1 (Fig 4A. *p* = .003 (LSD); Fig 4B. *p* = .002 (LSD)). Specifically, with 7 days of denervation, the increase in β1 and β5 was 600% and 500% in WT. In contrast, in L7M1, β1 and β5 content only increased by 300% and 200%, respectively. At day 14, no strain difference in β1 and β5 content was detected. With denervation, β2 content in both strains showed a time-dependent increase at day 7 and day 14 (Fig 4C, *p* < .001).

Fig 4D–4F highlights the LMP7, MECL-1, and LMP2 content in denervated muscles from WT and L7M1 mice. As expected, we did not detect any protein expression of LMP7 or MECL-1 in the L7M1 muscles (Fig 4D and 4E), which confirms the genetic knockout eliminated these subunits. In WT mice, LMP7 and MECL-1 content increased at day 7 and this increase was sustained through day 14 (Fig 4D and 4E). Lastly, LMP2 content, which is the only inducible subunit in L7M1 mice, increased in both strains following denervation. However, the WT showed a higher overall content compared to the L7M1 mice (Fig 4E, strain effect: *p* = .003).

In summary, in both strains, the subunits in the proteasome system, including α7, regulatory complexes, the standard β and the inducible subunits were elevated in the denervated muscle. However, the change in composition of the subunits in both strains did not parallel one another. Under the condition of denervation, there was a robust increase in β1 and β5 content in WT mice at day 7. However, the mice without immunoproteasome subunits LMP7 and MECL-1 had an attenuated response for β1 and β5. Also, lower content of LMP2 was observed in L7M1 muscles.

### Proteasome activities

**Fig 5:**
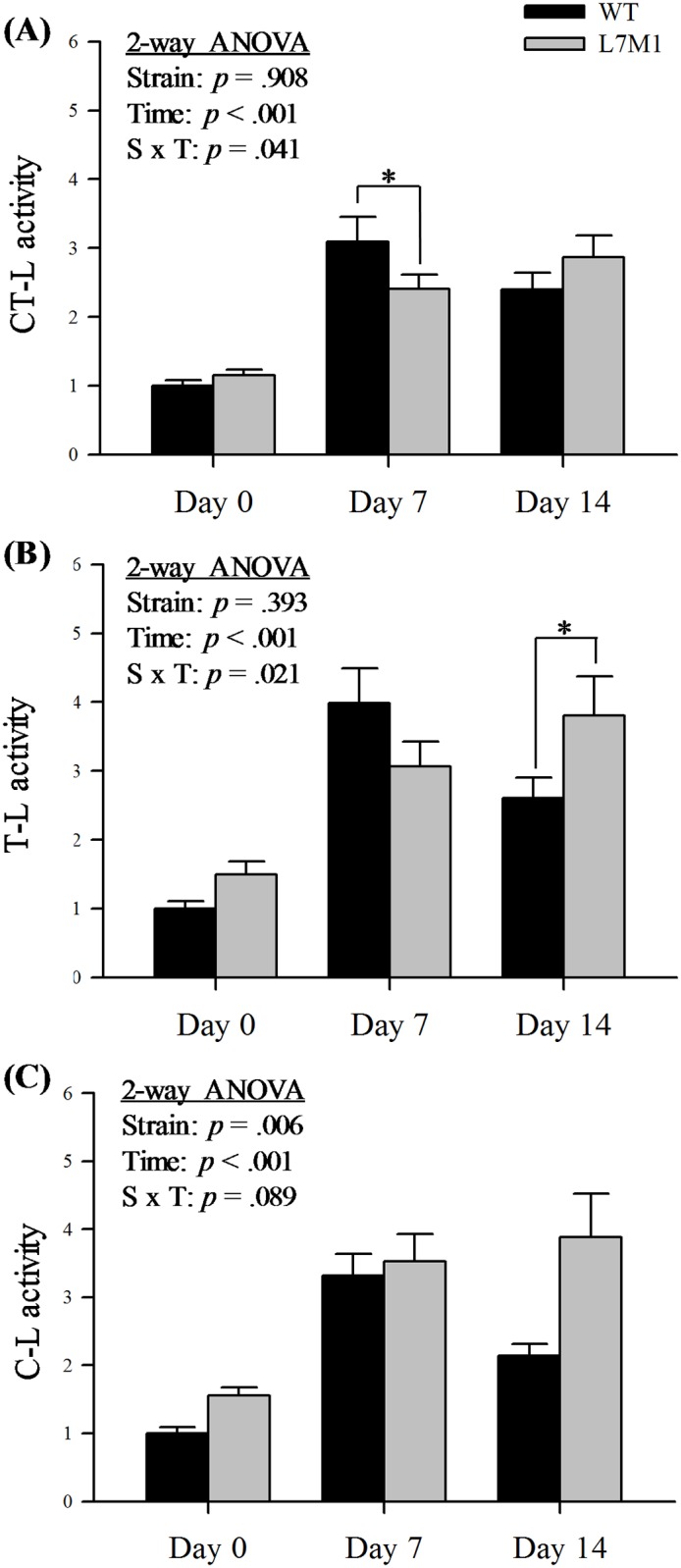
Proteasome enzymatic activities in WT and L7M1 mice with denervation. Results are presented as the fold change compared to WT Day 0 for proteasome enzymatic activity in each experimental group. Two-way ANOVA was used to detect the effect of strain (WT vs. L7M1) and time (Day 0, 7, and 14) for (A) Chymotrypsin- like (CT-L) activity, (B) Trypsin- like (T-L), and (C) Caspase- like (C-L) activity (# indicates significant effect of strain/time/S x T interactions, *p* < .05). When an effect of interaction was detected, a Fisher’s LSD *post-hoc* test was performed to compare differences between strains at each time point (* indicates L7M1 is significant different from WT at that given time point, *p* ≤ .05). Statistical comparison showed a significant denervation (time) effect in all proteasome activities (*p* < .001). No strain effect was detected in either CT-L or T-L activities; whereas the C-L activity showed significant strain difference (*p* = .01). (A) CT-L activity was significantly higher in the WT at day 7 compared to L7M1 (S x T, *p* = .04, LSD *post-hoc* test: *p* = .04). (B) T-L activity was significantly lower in the WT at day 14 compared to L7M1 (S x T, *p* = .02, LSD *post-hoc* test: *p* = .03). Values are mean ± SE. Sample size per group: n = 7–10.

To determine whether the genetic elimination of the two inducible subunits LMP7 and MECL-1 influences the function of the proteasome, the caspase-, chymotrypsin- and trypsin- like activities were monitored using fluorogenic peptide substrates with or without the proteasome inhibitor, MG132, to measure proteasome-specific activity (Fig 5). With denervation, both strains showed a significant increase in all three proteasome activities. The rate-limiting proteasome activity, chymotrypsin-like activity, showed an interaction between time and strain (Fig 5A, S x T: *p* = .041). Specifically, the chymotrypsin-like activity in WT mice was greater than the L7M1 mice after 7 days of denervation (Fig 5A, at day 7, WT *vs*. L7M1: *p* = .04 (LSD)). Similarly, an interaction was observed in the trypsin-like activity (Fig 5B, S x T: *p* = .021). At day 14, the trypsin-like activity in L7M1 mice was greater than the WT (Fig 5B, at day 14, WT *vs*. L7M1: *p* = .027 (LSD)). In contrast to the chymotrypsin-like and trypsin-like activities, the caspase-like activity did not show an interaction; however, there was a strain effect. Specifically, L7M1 mice showed greater caspase-like activity at baseline and across the denervation period compared to the WT (Fig 5C, strain effect: *p* = .001). Taken together, immunoproteasome deficiency altered the proteasome activities.

### Autophagy markers

**Fig 6:**
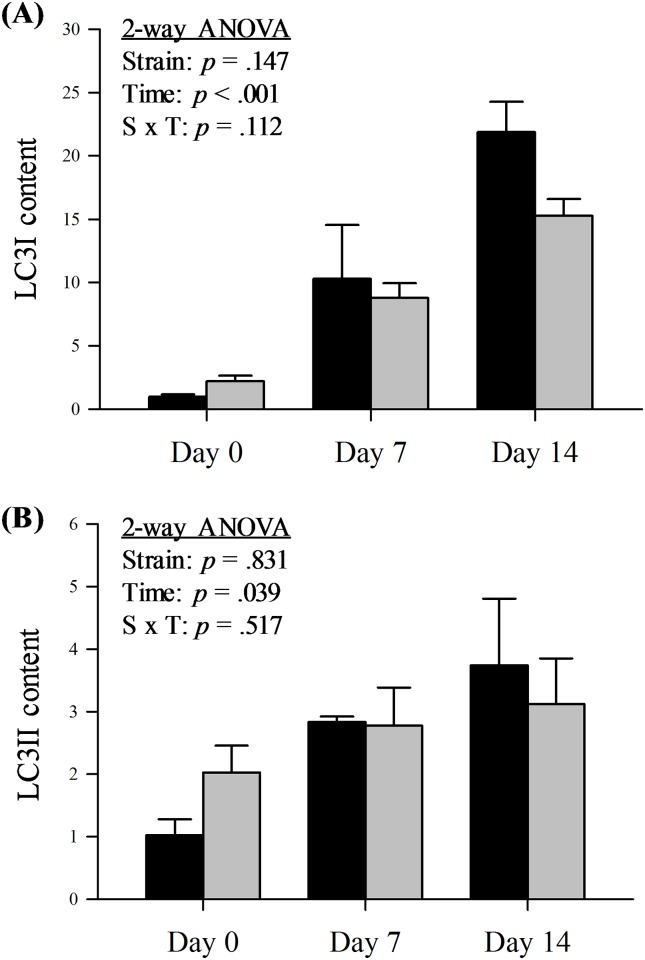
Protein content of autophagy markers LC3 in WT and L7M1 mice with denervation. (A-B) Results of densitometry are presented as the fold change compared to WT Day 0 for the autophagy marker LC3. Each panel contains results of two-way ANOVA that were used to detect the effect of strain (WT vs. L7M1) and time (Day 0, 7, and 14) for (A) LC3I and (B) LC3II (# indicates significant effect of strain/time/S x T interactions, *p* < .05). Statistical comparison showed a significant denervation (time) effect in both LC3I and LC3II content (*p* < .04). No strain effect or interaction was found. Values are mean ± SE. Sample size per group: n = 3–5. Representative Western blot for each protein is presented in Fig 1.

Autophagy is an alternative protein degradation pathway that is activated in denervation-induced muscle atrophy [26]. Although autophagic flux is the most accurate means to estimate the autophagy degradation activity, these measures require the application of lysosomal inhibitors, which cannot be done in whole muscle in a living animal [27]. However, Western blot analysis is still suitable to report the overall change of autophagic protein levels in a tissue, so we assessed the protein levels of the autophagic marker LC3 in the skeletal muscles from both strains of mice (Fig 6). During the process of autophagy, LC3I conjugates with phosphatidylethanolamine to convert to LC3II during the formation of the autophagosome [28]. These two species can be distinguished on Western blotting by their different mobility on an SDS-PAGE gel. We probed for both LC3I and LC3II content in order to estimate the activation of the autophagy pathway. The content of both LC3I and LC3II increased (Fig 6A and 6B) after denervation in both strains. These results indicate autophagy was activated in skeletal muscle and immunoproteasome deficiency did not alter the process of autophagy following denervation.

## Discussion

The purpose of this study was to determine if the immunoproteasome influences skeletal muscle proteolysis. To accomplish this, we denervated WT and L7M1 mice for up to 14 days and assessed the extent of muscle atrophy, proteasome content and proteolytic activity. From these experiments three important findings were noted. First, immunoproteasome content increased in response to denervation. Second, immunoproteasome deficiency affected specific catalytic sites of the proteasome following denervation, which changed the kinetics of proteasome activation. Lastly, in contrast to our hypothesis, immunoproteasome deficiency did not attenuate the loss of muscle mass at 7 or 14 days post-denervation. Taken together, these results suggest that the absence of immunoproteasome influences proteasome’s composition and function but does not appear to be essential for protein degradation during denervation-induced atrophy.

### Proteasome content following denervation

A significant time-dependent increase in content of standard proteasome was observed in both WT and L7M1 mice following denervation. In fact, α7, β1, β2, β5 and PA700 were all significantly greater at 7 and/or 14 days post-denervation when compared to control muscle (day 0). These results are consistent with that of others who demonstrated similar findings at the mRNA and/or protein level following 3, 7 and/or 14 days after denervation [5,11–14]. We also observed a significant increase in several components associated with the immunoproteasome. Specifically, PA28, MECL-1, LMP2, LMP7 all increased over 80% after 7 and 14 days of denervation in the WT mice. Although no study has yet to perform such a comprehensive examination of immunoproteasome content following denervation, work by Gomes *et al*. [11] demonstrated the protein content of MECL-1 was elevated ~ 2.3 fold in 14 day denervated muscle. Together, our findings confirm that the standard proteasome is upregulated in atrophying muscle but more importantly, demonstrate for the first time that all the immunoproteasome subunits also increase in response to denervation at the protein level for the first time.

### Incorporation of the proteasome subunits

There were some proteins in the L7M1 mice that did not increase to the same extent as in the WT mice. In particular, the catalytic subunits β1 and β5 were significantly lower 7 days post-denervation when compared to that of the WT muscle. It is possible that this blunted response is due to an impaired incorporation of these subunits into the 20S core of the standard proteasome [20,29]. In support of this, β5 was found to be more efficiently incorporated into the immunoproteasome when MECL-1 was present [30], while others have demonstrated that 20S assembly was decreased in LMP7 mutant cell lines [31]. Similar findings have been demonstrated in the spleen, retina [18,25], and C2C12 myoblasts [32], where decreased incorporation of LMP2 was noted with deletion or inhibition of LMP7 and/or MECL-1. Together, LMP7 and MECL-1 likely exhibit a role in facilitating the incorporation of the standard proteasome subunits, as well as the inducible subunit LMP2. Because we used an enriched-proteasome preparation, which only contains proteasome subunits fully incorporated into the mature 20S core [21,33], we are not able to quantify the proteasome subunits that are not incorporated into the 20S core.

### Proteasome proteolytic activity

The standard β and inducible subunits all perform distinct enzymatic activities to catalyze the digestion of proteins into peptides. Thus, any changes in subunit composition have the potential to influence and subsequently explain the enzymatic activity response. Chymotrypsin-like activity is orchestrated by β5, LMP2, and LMP7 while trypsin-like activity is regulated by β2 and MECL-1 and caspase-like activity is regulated by β1. Our results demonstrate all activities are increased for both WT and L7M1 mice following denervation, similar to that previously reported [11]. However, with the deletion of two inducible subunits, all three proteasome activities were altered post-denervation compared to the WT mice. The difference in kinetics of proteasome activation between strains was consistent with the findings in proteasome subunits after denervation.

The rate-limiting proteasome activity [34–36], chymotrypsin-like activity, was blunted in the immunoproteasome deficient mice at 7 days in contrast to the WT. This response is consistent with its subunit composition (lower β5 and LMP2 and no LMP7), which is likely due to the impaired incorporation of these subunits into the proteasome core [29,30], as mentioned above. Although LMP7 and LMP2 both contribute to chymotrypsin-like activity and their content is increased, their direct contribution to the overall proteolytic function is likely minimal due to two reasons. First, in un-manipulated skeletal muscle the immunoproteasome represents a small percentage (5%) of the total 20S proteasome [15]. Second, with denervation both standard and immunoproteasome content increase; however, the fold increase is greater in the standard subunits.

The caspase- and trypsin-like activities were also altered in the immunoproteasome deficient mice, though they have less importance in protein degradation than the chymotrypsin-like activity. For instance, inhibition of caspase-like or trypsin-like activity did not lead to dysfunction of protein degradation [34,37,38]. One interesting finding in the current study involves the time course of the caspase-like and the trypsin-like activities associated with denervation in the L7M1 mice. The content of β2, MECL-1 and β1 within each muscle does not appear to support the kinetic changes in the enzymatic activities. This unexpected finding, lack of a relationship between subunit composition and its activity, may involve the presence of intermediate proteasomes. The intermediate proteasome is a mixture of both standard and inducible subunits in the 20S core, where the inherent quality of the proteasome enzymatic activity is reported to be quite variable [15]. Therefore, the variability of the trypsin-like and the caspase-like activities in the current study may be due to presence of the intermediate proteasomes [15,20,25].

### Signaling pathways

Several major signaling proteins/pathways are known to up-regulate proteasome content. These include the mammalian target of rapamycin (mTOR) and the HDAC/Mitogen-activated protein kinase (MAPK). mTOR [39,40] is often identified as a negative regulator of the autophagy pathway [12,41], but also can increase expression of the standard proteasome subunits and PA700. In supports of this, inhibition of mTOR with rapamycin attenuated increases in standard proteasome subunits and the activator PA700 in the mouse liver [42]. The HDAC/Mitogen-activated protein kinases (MAPK) cascade is also known to influence proteasome content. In denervated muscle, HDAC4 triggers MAP3 kinase, which in turn regulates activator protein 1 (AP1) and specificity protein 1 (Sp1) [43,44], two transcription factors associated with LMP2, LMP7 and MECL-1 expression [45,46]. It is likely that the protein content of the standard proteasome and immunoproteasome increased following denervation due to changes in both mTOR and HDAC signaling.

### Atrophy and Autophagy

Despite the notable differences in the proteasome subunit content and activities between the two strains, the overall extent of muscle atrophy remained the same. It seems likely, even with the 25% lower rate-limiting chymotrypsin-like activity and the potential impaired proteasome assembly in the immunoproteasome deficient mice, lacking the two inducible subunits was not enough to affect proteasome-mediated proteolysis. It is possible that the actual impact of the immunoproteasome on muscle size is too small to detect under conditions of severe, rapid atrophy (i.e. denervation) due to the fact that it represents a low percentage of the total proteasome (5%) [15]. Consistent with others [13,14], we observed autophagy activation in the denervated WT mice. However, the denervated muscle from the WT and L7M1 did not differ in this autophagy activation, meaning that this proteolytic pathway, like the proteasome, remained intact. One additional point to be considered is that the rate and extent of atrophy is not only controlled by proteolysis but rather reflects the balance between protein synthesis and degradation. Since the current study did not evaluate protein synthesis, we can only speculate that differences in protein synthesis also contribute to our findings.

## Conclusions

The current study demonstrated that immunoproteasome content increased in response to denervation-induced skeletal muscle atrophy. Moreover, we also observed that genetic elimination of the two inducible catalytic subunits, LMP7 and MECL-1, altered the kinetic changes in chymotrypsin-like activity by potentially suppressing β5 and LMP2 incorporation into the 20S core. Despite these changes, L7M1 mice experienced the same extent of muscle atrophy as that of the WT mice. Taken together, these results indicate that although the immunoproteasome content is upregulated with denervation, while it does not appear to have a major role in the overall myofibrillar proteolysis that is associated with atrophy. It is possible that the immunoproteasome has a role in regulating changes in cell signaling that accompany denervation.
